# Application of Real Time loop mediated isothermal amplification (RT LAMP) as a point of care assay using dried blood spots for rapid detection of HIV infection

**DOI:** 10.1186/1471-2334-14-S3-O11

**Published:** 2014-05-27

**Authors:** Lavanya Vanjari, Lakshmi Vemu, Lalit Dandona, John A Schneider

**Affiliations:** 1 Department of Microbiology, Nizam’s Institute of Medical Science, Hyderabad, India; 2 Public Health Foundation of India, New Delhi, India; 3 University of Chicago, USA

## Background

Early and rapid diagnosis of HIV is essential to reduce the transmission and to start Anti Retroviral Therapy. The increasingly sophisticated molecular methods for diagnosing acute HIV infection have a direct impact on patient management as they have been developed to overcome the limitations of serological assays. Development of a further methodology, which is technologically simple and equally sensitive, accessible and affordable, is essential to detect the very few copies of viral genome during acute HIV infection. Hence in the present study a Real Time Loop Mediated Isothermal Amplification (RT LAMP) assay on Dried Blood Spots (DBS) for the detection of HIV RNA was developed for an early and specific diagnosis of HIV.

## Methods

To evaluate HIV RT LAMP in comparison with real time PCR, 600 DBS samples from high risk groups (300 truck drivers and 300 Men who have sex with men) and 100 healthy and age matched individuals (control group) were included in the study. Informed consent was obtained from all the patients and controls.

## Results

The performance characteristics of the HIV RT LAMP in comparison with Real Time PCRs were 100% sensitive and specific with a % CV less than 10.

## Conclusion

The HIV RT LAMP on DBS, developed in this study, proved to be a highly sensitive, accurate, simple and affordable assay and easy to be performed in any small laboratory, especially in developing countries with a potential of a point of care assay, costing approximately 5 times lesser than the sophisticated molecular methods.

